# Does COVID‐19 contribute to development of neurological disease?

**DOI:** 10.1002/iid3.387

**Published:** 2020-12-17

**Authors:** Arehally M. Mahalakshmi, Bipul Ray, Sunanda Tuladhar, Abid Bhat, Shasthara Paneyala, Duraisamy Patteswari, Meena Kishore Sakharkar, Hamdan Hamdan, David M. Ojcius, Srinivasa Rao Bolla, Musthafa Mohamed Essa, Saravana Babu Chidambaram, M. Walid Qoronfleh

**Affiliations:** ^1^ Department of Pharmacology, JSS College of Pharmacy JSS Academy of Higher Education & Research Mysuru Karnataka India; ^2^ Center for Experimental Pharmacology and Toxicology (CPT), Central Animal Facility JSS Academy of Higher Education & Research Mysuru Karnataka India; ^3^ Department of Neurology JSS Hospital Mysuru Karnataka India; ^4^ Division of Cognitive Neuroscience and Psychology, Faculty of Life Sciences JSS Academy of Higher Education & Research Mysuru Karnataka India; ^5^ The Drug Discovery and Development Research Group, College of Pharmacy and Nutrition University of Saskatchewan Saskatoon SK Canada; ^6^ Department of Physiology Al Faisal University Riyadh Saudi Arabia; ^7^ Department of Neuroscience Baylor College of Medicine Houston Texas USA; ^8^ Department of Biomedical Sciences, Arthur Dugoni School of Dentistry University of the Pacific San Francisco California USA; ^9^ Department of Biomedical Sciences, School of Medicine Nazarbayev University Nur‐Sultan 020000 Kazakhstan; ^10^ Department of Food Science and Nutrition, CAMS Sultan Qaboos University Muscat Oman; ^11^ Principal Investigator, Ageing and Dementia Research Group Sultan Qaboos University Muscat Oman; ^12^ Research & Policy Department World Innovation Summit for Health (WISH) Qatar Foundation Doha Qatar; ^13^ Research & Policy Division Q3CG Research Institute Ypsilanti Michigan USA

**Keywords:** ACE2, COVID‐19, glial cells, immune mediated demyelination, memory impairment, neurodegeneration, neuroinflammation, neurological circuits, neurology, SARS‐CoV‐2

## Abstract

**Background:**

Although coronavirus disease 2019 (COVID‐19) has been associated primarily with pneumonia, recent data show that the causative agent of COVID‐19, the coronavirus severe acute respiratory syndrome coronavirus 2 (SARS‐CoV‐2), can infect a large number of vital organs beyond the lungs, such as the heart, kidneys, and the brain. Thus, there is evidence showing possible retrograde transmission of the virus from the olfactory epithelium to regions of the brain stem.

**Methods:**

This is a literature review article. The research design method is an evidence‐based rapid review. The present discourse aim is first to scrutinize and assess the available literature on COVID‐19 repercussion on the central nervous system (CNS). Standard literature and database searches were implemented, gathered relevant material, and extracted information was then assessed.

**Results:**

The angiotensin‐converting enzyme 2 (ACE2) receptors being the receptor for the virus, the threat to the central nervous system is expected. Neurons and glial cells express ACE2 receptors in the CNS, and recent studies suggest that activated glial cells contribute to neuroinflammation and the devastating effects of SARS‐CoV‐2 infection on the CNS. The SARS‐CoV‐2‐induced immune‐mediated demyelinating disease, cerebrovascular damage, neurodegeneration, and depression are some of the neurological complications discussed here.

**Conclusion:**

This review correlates present clinical manifestations of COVID‐19 patients with possible neurological consequences in the future, thus preparing healthcare providers for possible future consequences of COVID‐19.

## Graphical abstract

This review correlates present clinical manifestations of coronavirus disease 2019 patients with possible neurological consequences. Recent studies suggest that activated glial cells contribute to neuroinflammation and the devastating effects of severe acute respiratory syndrome coronavirus 2 infection on the central nervous system.

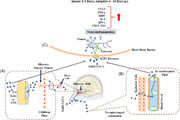

## 1 INTRODUCTION

Coronavirus disease‐19 (COVID‐19) was first identified in Wuhan, China, in early December 2019. In the past few months, this pandemic disease had spread all over the world and caused over 38,925,204 confirmed infections, with 1,098,378 fatal cases globally as of 16 October 2020, according to the Johns Hopkins University dashboard.

Coronaviruses (CoVs) are not new. The first described coronavirus was isolated from chickens in 1937. Human coronaviruses (HCoVs) were first found to be pathogenic in the mid‐1960s by Tyrrell and Bynoe.^1^, ^2^ HCoVs received more attention globally during the 2002‐2003 outbreak of severe acute respiratory syndrome (SARS) by SARS‐CoV, and the Middle East respiratory syndrome coronavirus (MERS‐CoV) outbreak in 2012. Until then, HCoV strains were only known to cause mild upper respiratory tract infections.

Infections by HCoVs (229E, OC43) are known to cause 15%–29% of common cold‐like conditions^3^ with mild upper respiratory infections. The epidemic of SARS‐CoV in 2002‐2003 showed their potential for high virulence. Since the SARS outbreak, five new HCoV strains (SARS‐CoV, NL63, HKU1, MERS‐CoV, and SARS‐CoV‐2) have been identified. Of these, NL63 and KHU1 cause mild upper respiratory tract infections, with fever and very few fatalities. SARS‐CoV, MERS‐CoV, and SARS‐CoV‐2 are highly contagious and pathogenic and cause lower respiratory tract infection in the elderly and in the immunocompromised.^4^ Interestingly, HCoVs like SARS‐CoV, MERS‐CoV, and SARS‐CoV‐2 have been reported to cause respiratory, enteric, hepatic and neurological disease, with variable clinical severity.^5^ This review is an attempt to gather data from isolated reports and elaborates on the potential of COVID‐19 to cause neurological complications like immune‐mediated demyelinating disease, cerebrovascular damage, neurodegeneration, and depression.

## 2 CORONAVIRUSES AND THE BRAIN

Reports from preclinical studies show that SARS‐CoV can access the brain through the olfactory bulb, and from there it reaches the brain via trans neuronal spread resulting in significant neuronal infection in SARS‐CoV‐receptor transgenic mice.^6^ Recent studies also show that SARS‐CoV‐2 is more transmissible than SARS‐CoV.^7^ The high homology between SARS‐CoV‐2 and the previous generations of SARS and MERS coronaviruses suggests that SARS‐CoV‐2 could potentially damage the neurological system.^8^

Genomic analysis reveals similarity between SARS‐CoV‐2 and SARS‐CoV, and this is consistent with the similarity in symptoms and pathogenesis of both viruses. SARS‐CoV and SARS‐CoV‐2 also have a high affinity for the angiotensin‐converting enzyme 2 (ACE2) receptor through which they can gain access to respiratory alveoli.^9^

The extent of SARS‐CoV‐2 infection of the brain might be influenced by various factors, including environmental and genetic. Some patients with COVID‐19 infection also have chronic diseases, which might have increased their risk to infection and decreased their immune‐mediated responses. Limited information is available on how this virus induces immunologic responses to infection in the brain or the related neuropsychiatric outcomes. Neural and immune cells serve as reservoirs of latent SARS‐CoV‐2, which may contribute to the delayed neurodegenerative events.^10^, ^11^

Interestingly, other than respiratory distress as a major symptom of COVID‐19, patients also experience headache, nausea, dizziness and vomiting, which suggests a probable involvement of the nervous system.^12^ Furthermore, out of 214 SARS‐CoV‐2 patients in one study, 78 patients had neurological symptoms. Severely sick patients exhibited neurological symptoms such as cerebrovascular disease, impaired consciousness and skeletal muscle injury.^13^ Reports also suggest that some SARS‐CoV‐2 coronavirus can also spread from lungs and lower respiratory tract to cardiovascular and respiratory centers in the medulla of the brain via mechanoreceptors and chemoreceptors through synaptic routes. The brain stem is the most affected SARS‐CoV‐2 target area of the brain in both experimental animals and patients.^8^

Although the ACE2 receptor is expressed by different tissues in the body, and the cells expressing ACE2 receptors are targets for the SARS‐CoV‐2 infection,^14^ their expression in the oral cavity and tongue creates a possible gateway to the organism.^15^ SARS‐CoV‐2 docking studies to ACE2 receptors revealed that lungs, heart, kidneys, intestines, brain and testicles are the major targets.^16^ In the brain, besides neurons, the ACE2 receptors are also present on glial cells.^17^ Thus, glial cells might be a route for SARS‐CoV‐2 infection of the brain. Gene sequencing of cerebrospinal fluid shows the presence of SARS‐CoV‐2, which adds to the evidence that the coronavirus has the ability to invade the central nervous system.^18^

In addition, studies have demonstrated increased expressions of ACE2 receptors in ischemic brains, diabetes and in smokers, suggesting increased susceptibility to SARS‐CoV‐2 infection.^19^ ACE2 null mice have been reported to show decreased amino acid tryptophan uptake from the gut, resulting in lower levels of the amino acid in the blood. It is speculated that this could possibly influence kynurenine pathway.^20^, ^21^ Upregulated or downregulated ACE2 receptors are expressed in many disease groups like depression, diabetes and ischemia. Hence, they are more vulnerable to infection by SARS‐CoV‐2.

Similarly, many reports describe central nervous system (CNS) infections by neurotropic viruses like cytomegalovirus, herpes simplex viruses, varicella‐zoster virus, West Nile virus (WNV), henipavirus, Japanese encephalitis virus, chikungunya virus, Ebola virus and rabies virus.^22^, ^23^ HIV‐1 can also cross the blood‐brain barrier (BBB) either paracellularly or transcellularly, and invade the CNS through a “Trojan horse” mechanism via the infected blood cells. Here, the infected monocytes cross the BBB via the production of pro‐inflammatory mediators like CCL2, which compromise the BBB.^24^ Rabies virus binds to nicotinic acetylcholine receptors at neuromuscular junctions, and travel into motor and sensory neurons.^25^ WNV is also reported to cause associated encephalitis by disrupting the BBB, and resulting in microglia activation, inflammation and loss of neurons.^26^

## 3 POSSIBLE ACCESS OF CORONAVIRUS TO THE NERVOUS SYSTEM

**Figure 1:**
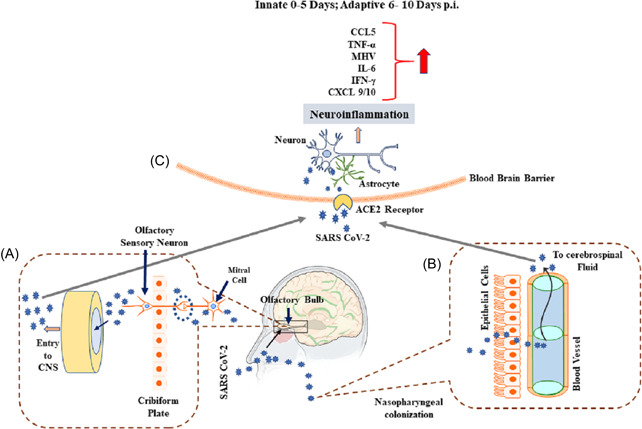
Possible entry routes of SARS‐CoV‐2 into brain. (A) Describes entry of SARS‐CoV‐2 from nasal epithelium to olfactory bulb entering CNS. (B) Explains entry of SARS‐CoV‐2 from the nasopharyngeal region to cerebrospinal fluid, thus gaining access to CNS. (C) Describes binding of SARS‐CoV‐2 to ACE‐2 receptors on the endothelial cells of BBB thus invading CNSand stimulating the cytokine storm by binding to ACE‐2 receptors on the glial cells and neurons. ACE‐2, angiotensin‐converting enzyme 2; BBB, blood‐brain barrier; CNS, central nervous system; COVID‐19, coronavirus disease 2019; SARS‐CoV‐2, severe acute respiratory syndrome coronavirus 2.

The possible mechanistic pathway of penetration of the coronavirus into the nervous system could be either hematological or through peripheral nerves.^27^ In the hematological route of entry, the coronavirus either enters the leukocytes and enters the blood stream or enters the blood through mucosa. Many viruses can breach the BBB. The BBB in the healthy state prevents the breach by pathogens. However, if there is immunosuppression and inflammation, invasion of viruses can occur. Clinical and experimental animal studies reports that the neuro‐invasive potential of coronavirus spread from the respiratory tract to CNS occurs via retrograde axonal transport from peripheral nerves, such as olfactory nerve or through the hematogenous pathway^28^ (Figure 1). Substantiating this, a recent report presents the magnetic resonance imaging of COVID‐19 patient brain, demonstrating anosmia.^29^

## 4 CORONAVIRUS INFECTION AND IMMUNE‐MEDIATED DEMYELINATING DISEASES

Brain autopsy of multiple sclerosis patients showed the presence of antibodies for coronavirus.^30^ The presence of coronavirus antigen and RNA in active demyelinating plaques also suggests the possible involvement of coronaviruses in the etiology and pathogenesis of multiple sclerosis.^31^ The neurovirulence of the coronavirus depends on its ability to induce proinflammatory signals from brain cells for the recruitment of blood derived inflammatory cells. Viruses with varying neurovirulence infecting brain astroglia and microglia cultures (primary brain immune cell cultures) show variable capability to induce proinflammatory cytokines like interleukin 2 (IL‐12), p40, tumor necrosis factor α (TNF‐α), IL‐6, IL‐15, and IL‐1beta in both astrocytes and microglia of mouse brain and spinal cords.^32^ Infection of the human astrocytic cell lines U‐373MG with the OC43 strain of human coronavirus led to increased transcription of IL‐6, TNF‐α, and MCP‐1, altered matrix metalloproteinases‐2 and 9 activity, and upregulation of nitric oxide production in both U‐373 cells and CHME‐5 human microglial cell lines.^33^ These results suggest a possible role of coronavirus‐induced glial cell mediated inflammation leading to conditions such as immune mediated demyelination of neurons. The 229‐E coronavirus strains could also infect fetal astrocytes, adult microglia, astrocytes and oligodendrocytes in cell culture.^34^ A unique domain at the N‐terminus of spike protein conferring its ability to bind to ACE2 receptors is absent in the 229‐E strain. However, C‐terminal parts of the spike protein in conjunction with specific amino acids bind to CD13.^35^

Experiments confirmed the ability of the coronavirus strain HCV‐OC43 to persistently infect astrocytic cell lines U‐373 MG and U‐87, suggesting the possible role of human coronaviruses to persist in CNS by targeting astrocytes.^36^, ^37^ Further intracerebral infection of rats with coronavirus results in initial downregulation of transcription of myelin protein roteolipid protein, leading to infected oligodendrocytes, followed by necrosis of the demyelinating lesions; whereas oligodendrocytes without detectable virus antigen levels were observed to undergo apoptosis. Although minimal remyelination was observed after clearance of virus antigen in oligodendrocytes, the destruction of oligodendrocytes continued due to apoptosis.^38^ Coronavirus‐induced encephalomyelitis in Lewis rats is reported to result in necrosis of infected oligodendrocytes followed by formation of demyelinated plaques. The central area of the plaques shows no virus antigen, while the peripheral regions of the plaques displayed virus antigen. Also, the virus‐induced inflammatory demyelination displayed increased expression of interferon γ (IFN‐γ), IL‐2, TNF‐α, iNOS and a novel cytokine, endothelial monocyte activating polypeptide II along with increase in the messenger RNA (mRNA) levels of regulatory calcium binding S_100_ proteins MRP8, MRP14, and CP10.^39^ Parra et al.^40^ reported that inhibition of IFN‐γ signaling reduces coronavirus replication; however, demyelination, axonal damage and infection of oligodendrocytes continued. Human coronavirus OC43 inoculation in mice resulted in infection of the CNS as a whole and the devastating effects of the virus were mostly attributed to the microglial reactivity and inflammatory reactions. Apparently microglial reactivity was due to direct neuronal injury.^41^

A murine coronavirus produced upregulation of Class I major histocompatibility complex antigens in oligodendrocytes and astrocytes. Induction of H‐2 antigen causes glial infection and triggers glial‐immune reactions.^42^ These data strongly suggest that the pattern of virus‐induced demyelination involves immune glial cells, which causes tissue destruction during the course of the disease. Indeed, the status of oligodendrocyte precursor cells, oligodendrocyte differentiation, axonal contact and myelin regeneration need to be studied further in coronavirus infections. Similar pathophysiological circumstances involving glial cells (being primary target) and oligodendrocytes, hence demyelination, may be expected in SARS‐CoV‐2 infection.

## 5 CORONAVIRUS AND CEREBROVASCULAR HEALTH

SARS‐CoV‐2 may reach the cerebral vasculature through the general circulation, possibly by breaching the BBB and affecting the parenchyma.^17^ A possible risk for stroke in respiratory virus infections was demonstrated by Warren‐Gash et al.^43^ One of the clinical studies reported four patients positive for stroke who were also positive for COVID‐19. All of the four cases displayed cerebrovascular accidents at early stages of illness.^44^ Interestingly, another study reported a 3% incidence of thrombotic complications in the COVID‐19 patients with critical illness.^45^ Oxley et al.^46^ reported five cases of large vessel stroke in COVID‐19 patients. Cases of large vessel strokes were also reported in the SARS‐CoV‐2 outbreak in Singapore.^47^ Reports propose that coagulopathy and vascular endothelial dysfunctions are also complications of SARS‐CoV‐2 infection.^48^ The etiopathological reasons for COVID‐19 induced stroke may range from inflammation induced venous and arterial thromboembolism, and hypoxia to diffused intravascular coagulation.^45^ A case study of a COVID‐19 patient reported ischemic stroke attributed to infection‐induced hypoxia and excessive secretion of inflammatory cytokines.^49^ Another study found higher *d*‐dimer or fibrin degradation product levels predisposing to a hypercoagulable state, and lower platelet count‐induced cerebrovascular hemorrhage in SARS‐CoV‐2 positive cases.^50^, ^51^, ^52^ Coagulopathy and antiphospholipid antibodies were also observed in critically ill COVID‐19 patients.^53^ A correlation was observed between cytokines released, encephalopathy and stroke symptoms in a COVID‐19 patient with cortical stroke.^54^ Reports show the ability of SARS‐CoV to induce polyneuropathy, encephalitis and aortic ischemic stroke.^55^ Data also shows influenza virus triggering a cytokine cascade and thereby exacerbating ischemic brain damage and intracerebral hemorrhage after treatment with tissue plasminogen activator.^56^ Interestingly, SARS‐CoV‐2 infections also result in cytokine storms.^57^ These similarities suggest that viral infection‐induced cytokine release mediated cerebrovascular dysfunctions may be one possible mechanism leading to stroke.^58^

## 6 CORONAVIRUS AND NEURODEGENERATION

Upregulation of the SARS‐CoV open reading frame, ORF‐6, leads to enhanced apoptosis via caspase‐3 mediated ER‐stress and JNK‐dependent pathways.^59^ SARS‐CoV ORF‐9b is localized on host cell mitochondria and disrupts mitochondrial functions to suppress host innate immunity.^60^ SARS‐CoV infection is also reported to induce mRNA levels of several UPR proteins like GRP78, GRP94, and C/EBP homologous protein, along with the accumulation of viral spike proteins in the endoplasmic reticulum.^61^ Coronaviruses affect some of the host proteases like endosomal cathepsins, cell surface transmembrane protease or serine proteases, furin, and trypsin.^62^ Most of these proteases are known for their involvement in the pathogenesis of various neurodegenerative diseases. Cathepsin D plays an important role in degrading altered neuronal proteins like alpha‐synuclein, amyloid precursor and huntingtin, whose abnormal degradation by altered protease could lead to accumulation of these proteins, which are prominent in neurodegenerative diseases like Parkinson's disease (PD) and Alzheimer's disease (AD).^63^ Some reports also show possible interactions of SARS‐CoV with the CNS, resulting in signs of PD.^64^ Also intracerebral injection of influenza virus A shows its virulent effect on substantia nigra and hippocampus, causing formation of Lewy body like structures and suggesting a role for this viral infection in neurodegenerative diseases.^65^ Furthermore, mice expressing Parkinson's disease linked to p.G2019s LRRK2 mutation exhibit reovirus‐induced encephalitis, resulting in increased mortality; and brains from these mice also show increased accumulation of alpha synuclein.^66^

The findings so far on SARS‐CoV‐2 infection show similarity with pathogenesis due to infection with SARS‐CoV and H1N1 in multiple aspects, including the effects on mitochondrial function, proteases, and ER stress responses. These pathways are strongly correlated with pathogenesis of various neurodegenerative diseases.

## 7 CORONAVIRUS‐INDUCED AMYLOID BETA AGGREGATION AND MEMORY LOSS

Emerging evidence indicates that MERS‐CoV and SARS‐CoV can promote neurological complications.^67^, ^68^ Neuronal death, especially in the medulla of mice infected with SARS‐CoV, has been reported.^6^ Respiratory syncytial virus (RSV) and herpes simplex virus type 1 (HSV‐1) trigger the accumulation of a distinctive protein corona in different biological fluids, which represents the initial phase of viral–host interactions. HSV‐1 infects peripheral sensory neurons.^69^ Several studies have reported that HSV‐1 contributes to the progression of AD.^70^ HSV‐1 infection has also been found to promote the deposition of neurotoxic amyloid beta (Aβ) in brains of infected mice.^71^ HSV‐1 DNA was found to be localized within Aβ plaques in AD patients.^72^ Similarly RSV have been found to accelerate the deposition of Aβ in mice.^73^ Infection with RSV and HSV‐1 demonstrated that viruses can physically act as nano‐surfaces capable of catalyzing amyloid nucleation, leading to accelerated fibril formation. Increased levels of Apo‐E, which is a well‐known risk factor for AD, have been observed in the HSV‐1 corona.^73^ Accumulation of Aβ plaques results in memory impairment and synaptic dysfunction.^74^ Accumulation of Aβ downregulates the expression of synapse associated proteins like synaptophysin, SNAP‐25, PSD‐95, and p‐GluR1 at Ser 845 in the mouse hippocampus. These proteins are necessary for maintaining the synapse and intercommunication between the neurons.^74^ Aβ‐induced synaptotoxicity may be critical in inducing memory dysfunction. Reduced synaptophysin, SNAP‐25, PSD‐95, and p‐GluR1 expression in the hippocampus is associated with cognitive dysfunction and memory loss in AD patients.^75^

## 8 CORONAVIRUS ISOLATION‐INDUCED ANXIETY AND DEPRESSION, AND RELATIONSHIP WITH SEROTONIN

Most viral infections begin in the peripheral tissues. Despite protective barriers and the immune systems, viruses can invade the CNS through the bloodstream or by infecting the nerves connecting to peripheral tissues.^76^ A recent study reported SARS‐CoV‐2 infection of the brain, causing disturbances in the mental health of the patients with COVID‐19.^77^ At the same time, an increase in symptoms like anxiety and depression were expected during the circumstances and restrictions of the global pandemic.

In terms of pathophysiology, a closely related coronavirus (SARS‐CoV) is reported to be neurotoxic and affect mental health.^78^, ^79^, ^80^ A HCoV strain (HCoV‐NL63) is also reported to be associated with a mood disorder.^81^ Thus, there is a possibility that SARS‐Cov‐2 could induce some of the neurotoxic states and symptoms of SARS‐CoV infection.

In a recent retrospective study of 214 COVID‐19 patients in Wuhan, China, 36%–45% reported CNS‐related disorders like dizziness, headache, loss of smell, impaired consciousness, loss of taste, and muscle pain.^13^ In another study involving 144 COVID‐19 patients in Wuhan, 34.72% and 28.47% of the patients reported to have symptoms of anxiety and depression, respectively.^82^ Furthermore, among the survivors of SARS infection, patients were reported to have persistent elevated stress, and over 64% of the survivors are reported to have a combination of stress, anxiety, and depression.^83^ But it is still not known if the symptoms can be attributed to the viral infections.

Many studies suggest that downregulation of serotonin (5HT) plays a significant role in conditions like depression.^84^, ^85^, ^86^ Other studies show that viral infection can lead to production of cytokines that impair neuronal firing, causing depression‐like symptoms.^87^ Summarizing the clinical symptoms reported in SARS virus infection, there is thus the possibility that SARS virus infection affected mood by altering the serotonin system. Hence, targeting the serotonin system could be considered as a potential option in therapies being developed for treating depression and anxiety induced by the COVID‐19 infection.

Moreover, the World Health Organization is concerned about the psychological impact of COVID‐19 on health workers, and people are anxious about the risk of infection and adapting to protective measures such as social isolation.^82^ However, there is concrete evidence showing that social isolation and loneliness are negatively correlated with mental health.^88^ In a study among 1210 cases from the general population in China, during the initial outbreak of COVID‐19, 16.5% reported severe depression, 28.8% anxiety, and 8.1% severe stress.^89^ The levels of anxiety and depression were also high among medical staff in Wuhan who treated the COVID‐19 patients, in comparison to healthy people.^90^ In another similar study in Wuhan, China, with 1257 responding doctors, 50.4%, 44.6%, 34.0%, and 71.5% reported symptoms of depression, anxiety, insomnia, and distress, respectively.^91^

## 9 PERSPECTIVE FROM THE NEUROLOGIST'S CLINIC

The impact of COVID‐19 is affecting all ages of life.^92^, ^93^, ^94^, ^95^, ^96^, ^97^ Both neurotropic and neuro‐invasive properties of SARS‐CoV‐2 infection are increasing. A broad spectrum of neurological manifestations including demyelinating, vascular and degeneration have been cited, making it imperative for clinicians to maintain a holistic approach in tackling the complications of COVID‐19. Cytokine‐mediated inflammation can cause both encephalopathy and stroke, along with altered protease‐mediated neurodegeneration and neurotransmitter alteration, resulting in depression and anxiety. Neurologists should be aware of the multitude of manifestations of this viral infection, which can manifest itself even in the absence of prominent respiratory symptoms, which are the primary reported diagnostic criteria used by most healthcare providers.

## 10 CONCLUSION

Extensive reports of the pathogenesis of SARS‐CoV‐2 infection present a complex picture of the etiological factors involved, the intricate causes of disease, and their consequences. It is noteworthy that although the major clinical manifestations of the disease involve the respiratory system, the key mediator of the pathogenesis is related to the immune system. Retrograde transmission of virus into the CNS is clear from the available literature. Hyper‐induction of chemokines and cytokines and a compromised cellular immune response caused by direct infection or indirect injury of immune cells in the CNS may contribute to COVID‐19‐related neurotropism. A compromised immune response may further lead to aggravation of SARS‐CoV‐2‐induced CNS disorders alongside respiratory distress. Advances made in our understanding of the pathology and pathogenesis of COVID‐19 could potentially serve as a guide for neurologists in the diagnosis, prevention, and treatment of post‐COVID‐19 neurological effects. As the world is still combating the pandemic, the present review provides neurologists some directions on treating the post‐pandemic effects.

## CONFLICT OF INTERESTS

The authors declare that there are no conflict of interests.

## AUTHOR CONTRIBUTIONS

Arehally M. Mahalakshmi, Bipul Ray, Sunanda Tuladhar, Abid Bhat, Shasthara Paneyala, and Duraisamy Patteswari performed literature research, gathered and analyzed information, and generated short preliminary write‐ups. Meena Kishore Sakharkar, Hamdan Hamdan, and David M. Ojcius provided research insight, content examination, and supported wide ranging aspects of the manuscript development process. Saravana Babu Chidambaram, Srinivasa Rao Bolla, Musthafa Mohamed Essa, and M. Walid Qoronfleh completed the conceptual work, framework, final draft write‐up, critical reading, and editing. All authors read and approved the final manuscript.

## Data Availability

Data sharing is not applicable to this article as no new data were created or analyzed in this study. All information generated or analyzed during this study are included in this published article.
